# Cellular memory of rapid growth is sensitive to nutrient depletion during starvation

**DOI:** 10.3389/fmicb.2022.1016371

**Published:** 2022-11-21

**Authors:** Spencer Cesar, Jiawei Sun, Kerwyn Casey Huang

**Affiliations:** ^1^Department of Microbiology and Immunology, Stanford University School of Medicine, Stanford, CA, United States; ^2^Department of Bioengineering, Stanford University, Stanford, CA, United States; ^3^Chan Zuckerberg Biohub, San Francisco, CA, United States

**Keywords:** stationary phase, environmental fluctuations, microfluidics, single-cell analysis, spent medium, anaerobic, respiration

## Abstract

Bacteria frequently encounter nutrient fluctuations in natural environments, yet we understand little about their ability to maintain physiological memory of previous food sources. Starvation is a particularly acute case, in which cells must balance adaptation to stresses with limited nutrient supply. Here, we show that *Escherichia coli* cells immediately accelerate and decelerate in growth upon transitions from spent to fresh media and vice versa, respectively, and memory of rapid growth can be maintained for many hours under constant flow of spent medium. However, after transient exposure of stationary-phase cells to fresh medium, subsequent aerobic incubation in increasingly spent medium led to lysis and limited growth when rejuvenated in fresh medium. Growth defects were avoided by incubation in anaerobic spent medium or water, suggesting that defects were caused by respiration during the process of nutrient depletion in spent medium. These findings highlight the importance of respiration for stationary phase survival and underscore the broad range of starvation outcomes depending on environmental history.

## Introduction

Natural environments are constantly in flux, hence bacteria face changes in many variables of physiological importance such as pH, osmolarity, and nutrients. Nutrient fluctuations are an omnipresent part of life in a host gut for enteric bacteria such as *Escherichia coli*. Decades of research have investigated how bacteria adapt to nutrient depletion and restoration, with specific transcriptional (Sharma and Chatterji, 2010) and post-transcriptional (Iyer et al., 2018) responses in stationary phase. Recent studies have revealed physiological and morphological changes associated with stationary phase (Shi et al., 2021b; Cesar et al., 2022), and that the lag phase associated with rejuvenation from stationary phase upon exposure to fresh nutrients can be heterogeneous and associated with large-scale remodeling of cell shape (Shi et al., 2021a). However, most studies of lag and stationary phase have focused on single transitions, despite the fact that nutrient concentrations can fluctuate on a variety of time scales due to environmental factors such as diurnal cycles and host factors such as feeding and circadian rhythms.

During any transition between nutrient conditions, there is an inevitable period of adaptation. Growth kinetics can be suboptimal due to issues addressing metabolic bottlenecks (Erickson et al., 2017). A recent metabolomics study revealed that the long lags resulting from switches between certain carbon sources are due to the depletion of key metabolites that follows sudden reversal in central carbon flux (Basan et al., 2020). Thus, cells can be trapped in suboptimal growth states depending on their proteomic and metabolic history. A separate study repeatedly switched *E. coli* between glucose and lactose to investigate how long the memory of lactose persists in its absence, using growth rate to determine the extent of adaptation because growth rate transiently decreased when cells were maladapted to utilization of lactose (Lambert and Kussell, 2014). The extent and duration of memory was related to the abundance of the lactose permease LacY, and loss of memory resulted simply from dilution (Lambert and Kussell, 2014). These experiments suggest that proteome reallocation is important for adapting to nutrient shifts, and that the ability of cells to reprogram may be dependent on their ability to dilute certain components through growth. Consistent with this picture, during emergence from stationary phase, a model incorporating a time delay between the synthesis of cytoplasmic and surface-related proteins quantitatively predicted the dynamics of cellular dimensions, and inhibition of translation (presumably delaying proteome reallocation) increased the delay (Shi et al., 2021a). During repeated fluctuations between environments with different nutrient qualities, *E. coli* cells adapted to respond to the nutrient shifts at the cost of the overall growth rate (Nguyen et al., 2021). Switching to nutrient-depleted conditions in which growth rate is at or near zero has not been interrogated in these contexts.

Most investigations into physiology during starvation have focused on long time scales after gradual entry into stationary phase. *E. coli* cells remain metabolically active for long periods of time in stationary phase (Gefen et al., 2014), and *Bacillus subtilis* cells exhibit very slow growth and division long after entry into stationary phase (Gray et al., 2019). *E. coli* cells kept in stationary phase for many days acquired mutations, particularly in *rpoS*, that allowed them to survive harsh conditions that would otherwise cause death (Zambrano and Kolter, 1996; Farrell and Finkel, 2003) but resulted in maladaptation to growth in fresh medium (Zambrano and Kolter, 1996). Populations kept in stationary phase for shorter periods (such as a typical overnight laboratory culture) exhibit lag times on the scale of hours before reaching maximal growth rate. At the cellular level, it was recently discovered that rejuvenation is heterogeneous, such that the lag times of single-cell growth were distributed with a very long tail (Simsek and Kim, 2019). The distribution of single-cell lag times was dependent on the time spent in stationary phase (Cesar et al., 2022), and was due to the progressive accumulation of damage (Pu et al., 2019; Cesar et al., 2022). Importantly, incubation in stationary phase in an anaerobic chamber avoided delays in growth unless cells were provided nitrate as a source of respiration (Cesar et al., 2022), indicating that the relevant damage was induced by respiration (Pu et al., 2019). When exponentially growing *E. coli* cells are rapidly switched to nutrient-depleted conditions such as M9 salts or spent medium, a variety of phenotypes can emerge. In wild-type *E. coli*, the cytoplasm gradually shrank away from the cell wall, with a commensurate increase in the size of the periplasm (Shi et al., 2021b). In a dominant-negative mutant allele of *mlaA*, which encodes a component of the anterograde phospholipid transport machinery (Malinverni and Silhavy, 2009), a rapid switch to spent medium led to cytoplasmic shrinkage and subsequent cell lysis (Sutterlin et al., 2016; Grimm et al., 2020). This diverse range of cellular behaviors makes it challenging to predict the extent to which bacterial cells can survive and/or robustly regrow after exposure to spent medium, particularly over the course of multiple transitions.

Here, we show that the instantaneous growth rate of *E. coli* cells responds immediately to a switch from spent to fresh media, or vice versa. During repeated transitions between spent and fresh media, we demonstrate that cells can retain memory of their growth state in the most recent exposure to fresh nutrients. In microfluidic flow cells, memory was eventually lost during incubation in spent medium, but only after many hours. In stark contrast, transient exposure of stationary-phase cells to fresh medium in test tubes before resuspension in spent medium resulted in highly detrimental consequences, with slower growth upon rejuvenation, cell lysis, and some cells oscillating between growth and shrinkage. We discovered that growth defects did not occur when cells were resuspended in anaerobic spent medium or in water, suggesting that respiration as cells consume nutrients in spent medium is the cause of lysis. These findings highlight the wide range of physiological states after starvation, implicating complete nutrient depletion and respiration as major drivers of negative outcomes.

## Results

### Switching between spent and fresh media results in an immediate growth-rate response

**Figure 1:**
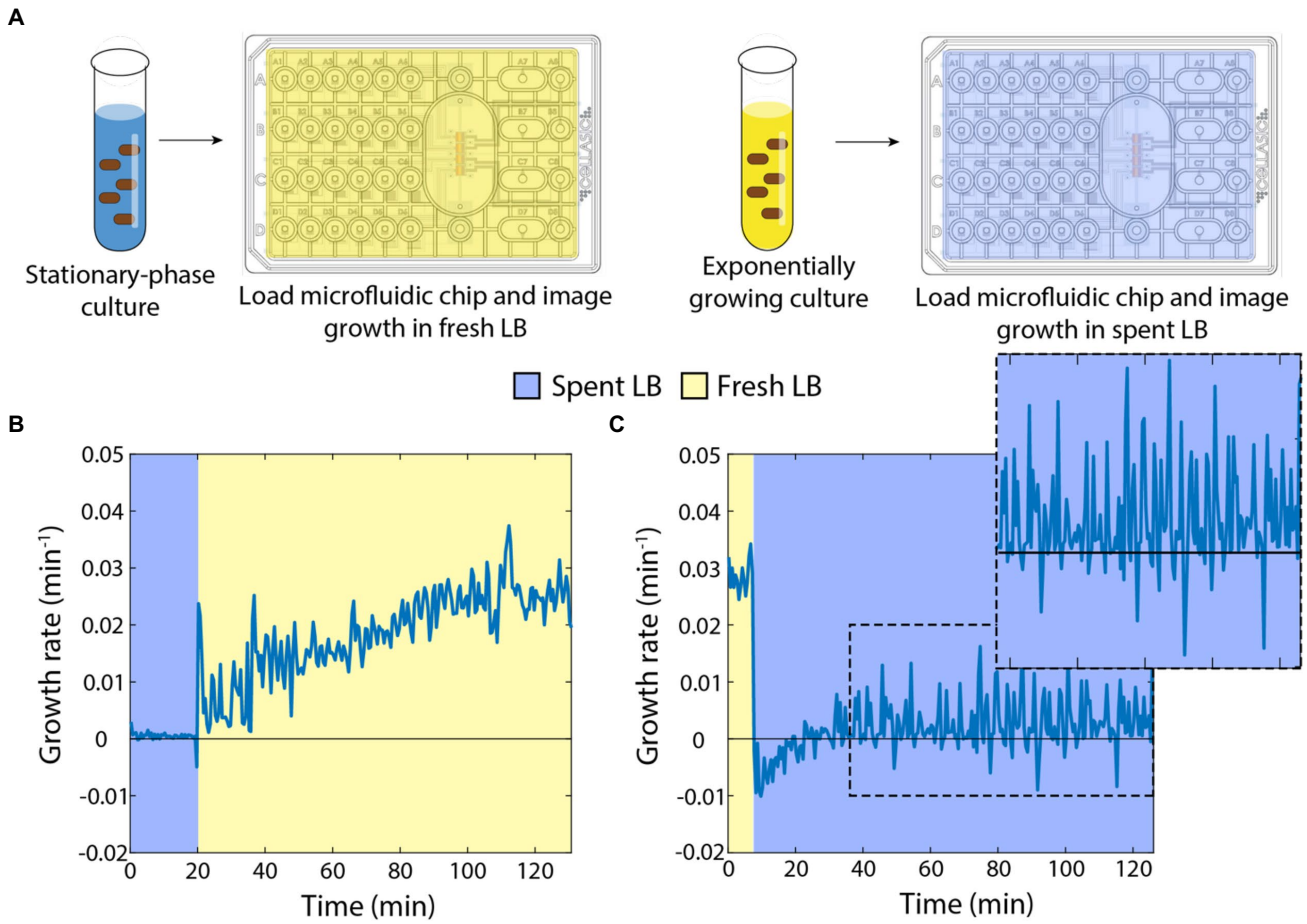
Switches between spent and fresh media result in almost immediate growth-rate responses. **(A)** Schematic of protocol for time-lapse imaging of *E. coli* in microfluidic flow cells during media-switching experiments. **(B)** Upon exposure to fresh LB, stationary-phase cells exhibited an initial increase in growth rate followed by gradual acceleration up to a maximum of ~0.03 min^−1^ over ~120 min. Curve is the mean growth rate for *n* > 80 cells, with similar behavior in ≥5 replicate experiments. **(C)** Exponentially growing cells halted their growth almost immediately after being switched to spent supernatant from a stationary-phase culture. Instantaneous growth rate was initially slightly negative but was slightly positive after ~30 min (zoomed inset). Curve is the mean growth rate for *n* > 80 cells, with similar behavior in ≥5 replicate experiments.

In previous studies, we showed that stationary-phase *E. coli* cells exhibit a rapid (within minutes if not less) increase in growth rate when placed on an agarose pad with fresh medium (Cesar et al., 2022), and that the growth of exponentially growing cells slows dramatically on agarose pads with spent supernatant (Sutterlin et al., 2016). To more precisely quantify the dynamics with which *E. coli* cells in either a fast-growing or starved state equilibrate to nutrient-poor or nutrient-rich media, respectively, we used a microfluidic flow cell (Figure 1A; Section “Materials and methods”) to track single cells during a nutrient transition. Microfluidic flow cells have been used as novel probes of cellular physiology, by enabling tracking of single-cell growth during rapid and repeated switching between environments (Rojas et al., 2014).

First, we grew an overnight culture in LB for 17 h, then spun down the cells to filter the supernatant. We incubated the starved cells in a microfluidic flow cell in spent medium and observed little to no growth for 20 min, as expected (Figure 1B). We then switched the medium to fresh LB and observed a rapid increase in growth rate within 1 min (Figure 1B). Thereafter, the instantaneous growth rate (defined here as 1/*A dA*/*dt*, Section “Materials and methods”) gradually increased to a peak of ~0.025–0.03 min^−1^ (doubling time of ~23–28 min), consistent with bulk culturing (Atolia et al., 2020). These data indicate that even after hours of starvation, cells are primed to restart growth.

Next, we used multiple 1:10 dilutions to establish cells in steady-state, exponential growth (Shi et al., 2017). We incubated these cells in a microfluidic flow cell with fresh LB and observed maintenance of rapid growth at a constant rate of ~0.03 min^−1^ (Figure 1C). After 20 min, we switched the cells into spent medium from a 17-h culture. Cells slowed to near zero growth rate almost immediately, and mean instantaneous growth rate even became slightly negative (Figure 1C), corresponding to cells shrinking in volume. Interestingly, after ~20 min, cell growth resumed, albeit at a very slow rate (Figure 1C, inset). Therefore, the transition to spent medium has two phases: an immediate cessation of growth and slow shrinkage, followed by adaptation to spent medium that allows for slow cell expansion on longer (hour) time scales.

### Cells can maintain memory of rapid growth during incubation in spent medium

**Figure 2:**
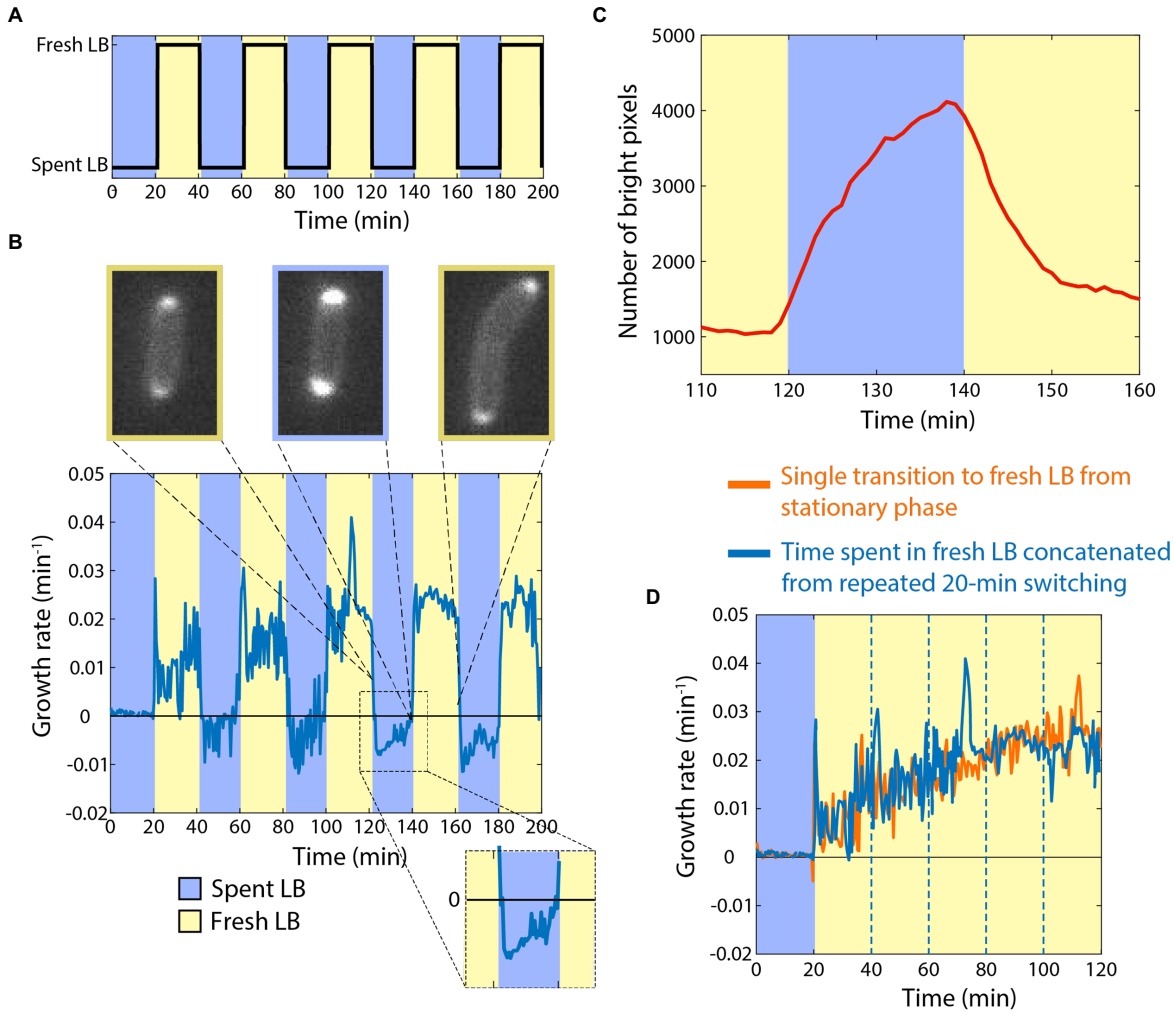
Cells can retain memory of their previous growth state during exposure to spent medium. **(A)** Dynamics of periodic switching between spent and fresh media using a microfluidic flow cell. **(D)** For switches every 20 min, growth accelerated during periods in fresh LB (yellow), and rapidly halted in spent LB (blue). During shifts to spent LB, growth rate was slightly negative (inset), and periplasmic mCherry accumulated at the poles. Curve is the mean growth rate for *n* > 180 cells, with similar behavior in ≥5 replicate experiments. **(C)** mCherry accumulated in the periplasm during the interval in spent medium (blue), and then immediately began to redistribute after switching to fresh LB (yellow). Shown is the number of bright pixels, defined as having intensity >350, with in the frames of the time-lapse imaging dataset in **(B)**; polar accumulation corresponds to a larger number of bright pixels. The curve was smoothed over 5 time points. **(C)** Aside from the peaks directly after switching to fresh LB, the dynamics of growth during intervals in fresh LB (blue, data from **(B)** with the intervals in spent LB extracted computationally) followed a quantitatively similar trajectory as cells that had not experienced switches back to spent LB (orange, data from Figure 1B).

Armed with knowledge of the baseline response of cells to the addition or removal of nutrients, we next sought to determine how cells would respond to periodic switching between rich and spent media (Figure 2A). As before, we started by incubating stationary-phase starved cells in spent medium, and then switched them to fresh LB for 20 min. As expected, the growth rate trajectory (Figure 2B) closely matched that of the single transition from spent to fresh medium (Figure 1B). We then switched cells back to spent medium, and observed rapid growth inhibition (Figure 2B), again as expected (Figure 1C). Upon a second switch to fresh LB, cells now resumed growth at a faster rate than upon the first switch to fresh LB, initially mirroring the growth rate achieved just before the previous transition to spent medium (Figure 2B). Growth acceleration continued for 20 min in fresh LB, and then rapidly halted upon switching back to spent medium (Figure 2B). A third switch to fresh LB again resulted in growth resuming at the rate cells achieved directly before the previous switch to spent LB (Figure 2B).

During the periods of incubation in spent medium, cells exhibited a slightly negative growth rate (Figure 2B), similar to the single transition from exponential growth to spent medium (Figure 1C). To interrogate this shrinking behavior, we repeated the experiments with a strain expressing cytoplasmic GFP and periplasmic mCherry. During switches from fresh to spent LB, cells accumulated periplasmic mCherry near the poles (Figure 2B,C; Supplementary Movie S1; Section “Materials and methods”), indicating that the cytoplasm had shrunk away from the cell wall in these locations. During the switches from spent to fresh LB, the periplasmic accumulation of mCherry quickly dissolved (Figure 2C; Supplementary Movie S1), which was coincident with the short (<1 min) bursts of faster growth upon transitions to fresh LB (Figure 2B). Thus, we infer that shrinkage and the bursts of growth were due at least in part to water efflux from and influx into, respectively, the cytoplasm.

We computationally extracted and concatenated the periods of growth in LB (Figure 2D) and compared to our single-transition data (Figure 1B). The growth rate trajectories were virtually identical aside from the short bursts after the transitions from spent to fresh LB, indicating that cells maintained near-perfect memory of the previous rapid growth state while incubating in spent medium, at least on the 20-min time scale.

## Memory of rapid growth decays slowly during microfluidic incubation in spent medium

**Figure 3:**
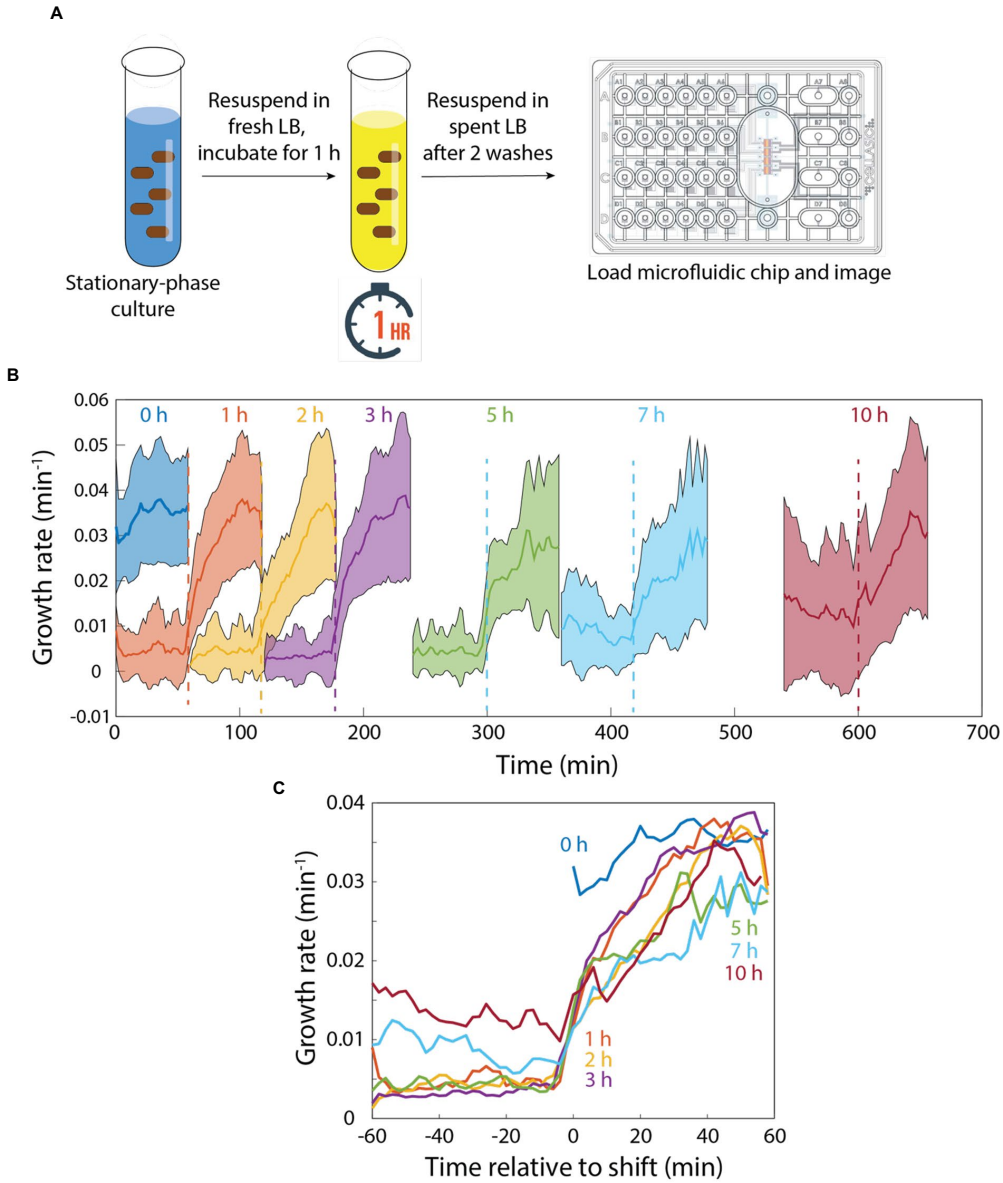
Memory of growth in fresh medium degrades slowly in perfusing spent medium. **(A)** Schematic of protocol for quantifying the response to pulses of fresh and spent medium. In a microfluidic flow cell, stationary-phase cells were diluted into fresh medium for 1 h, then switched to spent medium. **(B)** After various intervals, cells were switched to fresh LB. Growth was monitored before and after the switch back to fresh LB. **(B,C)** After 1 h of incubation in spent medium, growth trajectories upon switching to fresh LB were slightly slower than with no exposure to spent medium (0 h). The lag in regrowth increased by ~10 min for 2–5 h of incubation in spent medium. After 7 or 10 h of incubation in spent medium, growth rate was substantially increased in spent medium. While there was a further delay in rejuvenation after 7 h of incubation in spent medium, in all cases regrowth was faster than for a switch from stationary phase to fresh medium (Figure 1B). Curves in **(B)** are mean values and shaded regions represent 1 standard deviation. Curves in **(C)** are the same as in **(B)** except shifted to overlay the time at which cells were exposed to fresh LB. Each curve is the mean of *n* > 15 cells, and shading represents 1 standard deviation.

Since memory was maintained during 20 min of incubation in spent medium (Figure 2D), we next sought to determine the time scale over which memory would degrade. To address this question, we diluted a stationary-phase culture into fresh LB for 1 h, filtered the cells from their supernatant, and resuspended them in spent supernatant (Figure 3A). We then incubated the cells in spent medium in a microfluidic flow cell for intervals between 0 and 10 h before switching back to fresh LB (Figure 3A). After 0 h (continued growth in fresh LB), instantaneous growth rate was maintained at >0.03 min^−1^ (Figure 3B). After 1 h in spent medium, growth rate lagged behind the 0 h trajectory for the first 20 min, after which growth rate was >0.03 min^−1^ (Figures 3B,C). After 2–5 h in spent medium, growth rate lagged ~10 min behind the 1 h trajectory and plateaued at a slightly lower level after 60 min (Figures 3B,C). While cells that had been incubated for 1–5 h in spent medium exhibited little to no growth prior to the switch to fresh LB, after 7 or 10 h of incubation in spent medium instantaneous growth rate was ~0.01 min^−1^ (Figures 3B,C), potentially due to adaptation to the low, steady supply of nutrients in the spent medium (cannibalism could also contribute, although the constant perfusion of spent medium would likely wash away most of the contents of lysed cells). After the switch to fresh LB, cells that had been incubated for 7 h in spent medium exhibited slower growth than those incubated for 2–5 h (Figures 3B,C). Cells that had been incubated for 10 h in spent medium exhibited comparable growth to those incubated for 2–5 h, potentially due to the higher growth rates supported prior to switching (Figures 3B,C) that reduced the need for adaptation. Thus, cellular memory does eventually degrade in spent LB, but only over several hours, and even after 10 h growth rate acceleration was faster than after a transition of stationary-phase cells directly into fresh LB (Figure 1B), indicating that incubation in spent medium in a microfluidic flow cell did not require the same adaptation as long periods in stationary phase and that memory persists after very long exposure to low nutrient conditions.

### Lack of continuous flow severely impacts memory of fast growth

**Figure 4:**
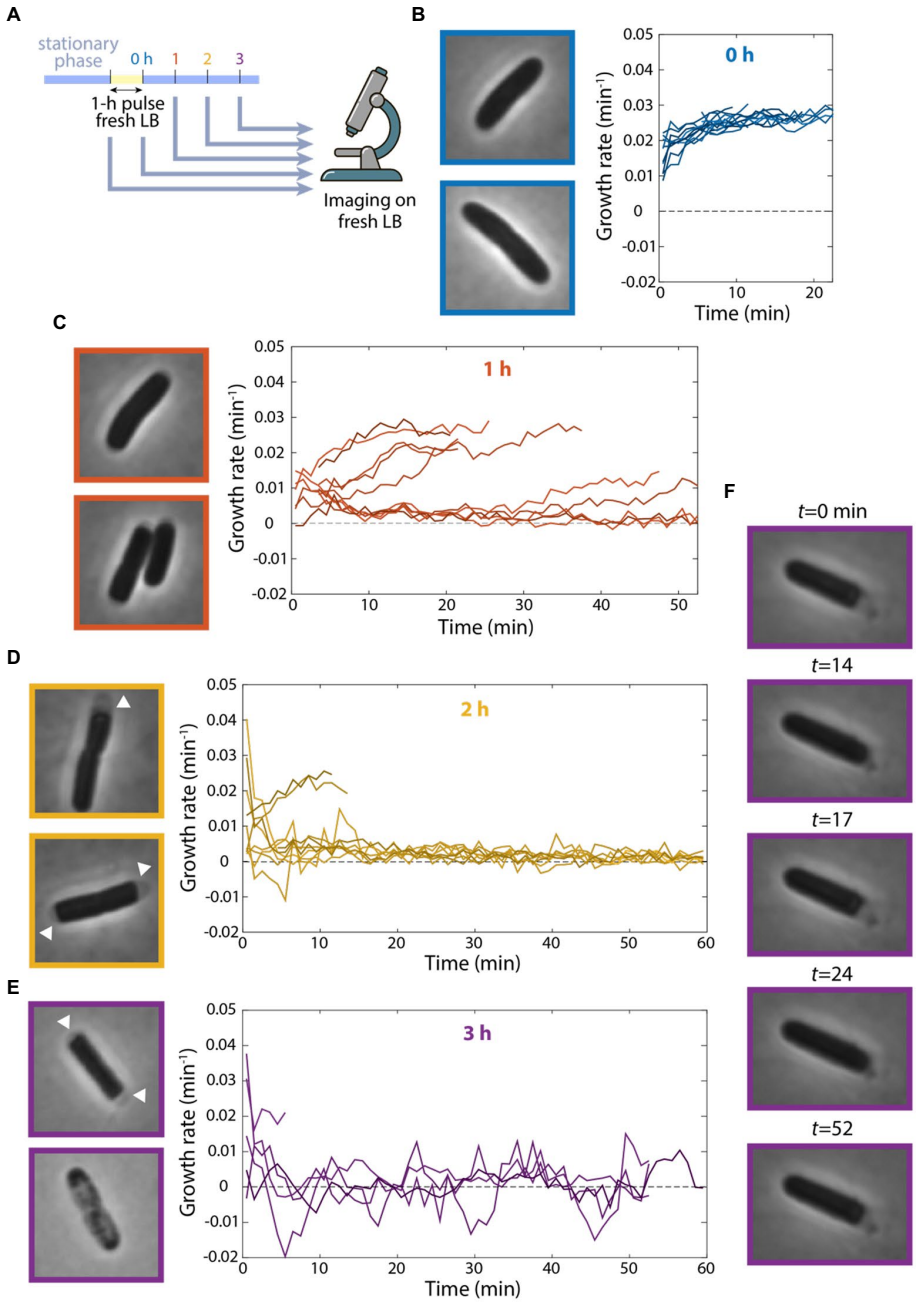
Continuous flow of spent medium is necessary for maintaining viability after transient exposure to fresh medium. **(A)** Schematic of experiment in which stationary-phase cells were diluted into fresh LB for 1 h and resuspended in spent supernatant for various intervals. Growth was then measured on agarose pads made with fresh LB via single-cell time-lapse imaging. **(B–E)** Cells showed growth defects after incubation in spent medium in a test tube. Left: images of cells at the start of imaging. Right: quantification of growth-rate dynamics for a subset of cells. *n* ≥ 35 cells were analyzed for each time point. Results were similar in a replicate experiment. **(B)** After 0 h (no resuspension in spent medium), cells grew at their maximal growth rate of ~0.03 min^−1^. **(C)** After 1 h of incubation in spent medium, growth rate for many cells was substantially lower than in **(B)**. **(D)** After 2 h of incubation in spent medium, growth rate was close to zero for most cells, and many cells exhibited cytoplasmic shrinkage (arrowheads). **(E)** After 3 h of incubation in spent medium, most cells exhibited cytoplasmic shrinkage (top cell) or had lysed at the start of imaging (bottom cell). **(F)** After 3 h of incubation in spent medium, one of the few cells that grew exhibited fluctuations between growth and shrinking.

The observation that cells could grow at a considerable rate after 10 h of incubation in spent medium in a microfluidic flow cell was surprising, so we next sought to replicate our microfluidic incubation experiments in bulk cultures. As before, we diluted a stationary-phase culture into fresh LB for 1 h, filtered cells from their supernatant, and resuspended the cells in spent medium. We sampled cells from the test tube after various intervals incubated in spent medium and imaged their growth on agarose pads with fresh LB (Figure 4A). Without resuspension in spent medium, cells quickly accelerated to a growth rate of ~0.03 min^−1^ (Figure 4B), as expected based on their maximum growth rate in exponential phase (Figure 1B). However, incubation in spent medium in a test tube radically altered single-cell behaviors. After 1 h of resuspension in spent medium, the growth rates of many cells were substantially (~3- to 4-fold) lower than exponentially growing cells (Figure 4C). Thus, resuspension in fresh medium in a test tube results in deleterious effects on growth.

After 2 h in spent medium, many cells displayed marked signs of cytoplasmic shrinkage at the start of imaging (Figure 4D), suggesting full depletion of nutrients (Shi et al., 2021b), unlike cells that had not been incubated in spent medium (Figure 4B). Moreover, many single-cell growth rates decreased drastically to nearly zero (Figure 4D). After 3 h in spent medium, most cells had lysed by the start of imaging and most of the remaining cells exhibited almost no growth (Figure 4E). To test whether cell lysis occurred prior to imaging or when cells were introduced to fresh medium on the agarose pad, we placed cells after 3 h in spent medium on agarose pads made with spent LB. We observed similar proportions of lysed cells (data not shown), suggesting that lysis resulted during spent medium incubation (unless they lysed immediately upon contact with the pad). Note that we rarely observed lysis in cells during stationary phase, even after 24 h, indicating that the 1-h pulse of fresh medium was the cause of lysis.

Interestingly, of the few cells that remained viable after 3 h in spent medium, several exhibited bizarre oscillations of growth and shrinking throughout the 55 min of tracking (Figure 4F; Supplementary Movie S2). These data show that incubation in a test tube, which lacks the continuous replacement of the contents of spent medium occurring in a microfluidic flow cell, leads to a dramatic decrease in growth rate and viability. Moreover, these findings indicate that transient exposure to fresh medium can have negative impact on growth potential.

### Cell lysis and growth slowdown results from depletion of nutrients in spent medium

**Figure 5:**
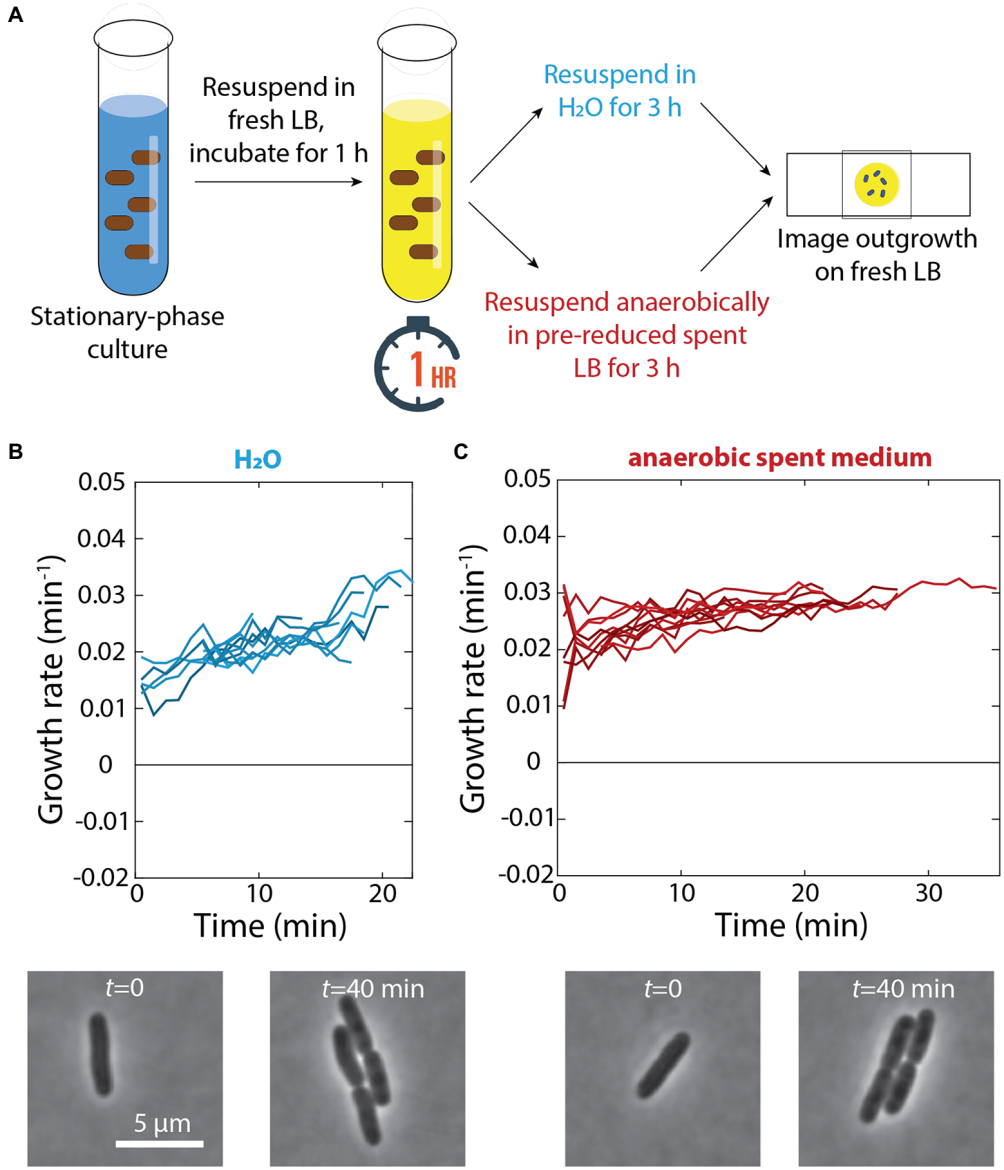
Incubation in water or anaerobic incubation in spent medium does not lead to growth defects. **(A)** Schematic of experiment in which stationary-phase cells were diluted into fresh LB for 1 h and resuspended in either H_2_O or anaerobically in pre-reduced spent medium for 3 h. Growth was then measured via single-cell time-lapse imaging on fresh LB agarose pads. **(B)** Top: After 3 h of incubation in water, when placed on agarose pads made with fresh LB cells did not exhibit any lysis and resumed rapid growth. Shown are growth-rate dynamics for a subset of cells. *n* > 50 cells were analyzed for each time point, and similar results were observed in a replicate experiment. Bottom: representative phase-contrast images illustrating growth. **(C)** Top: Aerobically grown stationary phase cells were exposed to a 1-h pulse of fresh LB and then resuspended in pre-reduced spent medium isolated from an aerobically grown culture. After 3 h of anaerobic incubation in the spent medium, cells were able to regrow rapidly. Shown are growth-rate dynamics for a subset of cells. *n* > 50 cells were analyzed for each time point, and similar results were observed in a replicate experiment. Bottom: representative phase-contrast images illustrating growth.

Our observation that cells adapted to grow very slowly in spent medium (Figure 1C) suggested that there are nutrients remaining in seemingly spent medium that can enable growth. Thus, we hypothesized that the growth defects of cells exposed to a 1-h pulse of fresh LB and then resuspended in spent medium in a test tube were due to cells with the physiology of rapid growth depleting the remaining nutrients in spent medium in the test tube and thus lacking the ability to deal with cellular damage. By contrast, switching from fresh to spent medium in a microfluidic flow cell would lead to relatively benign consequences since the spent medium (and the nutrients therein) was constantly being replenished. To test this hypothesis, we resuspended cells in ddH_2_O rather than spent medium after a 1-h pulse in fresh LB (Figure 5A); we did not observe any growth on agarose pads made with water over 4 h (data not shown). After 3 h of incubation in water following the 1-h pulse, cells did not exhibit any apparent lysis and resumed rapid growth when placed on LB pads (Figure 5B). Thus, we conclude that growth in spent medium is ultimately detrimental if cells run out of resources.

### Respiration may be the cause of growth defects after incubation in spent medium

Based on our previous discovery that cells no longer exhibit dormancy during emergence from stationary phase when respiration in stationary phase is prevented (Cesar et al., 2022), we hypothesized that the availability of oxygen caused growth slowdown and lysis after a pulse of fresh medium and subsequent incubation in spent medium. To specifically address the role of respiration in stationary phase (rather than changes in log-phase growth aerobically versus anaerobically), we resuspended aerobically grown cells in spent medium isolated from a culture grown aerobically the day before that had been reduced in an anaerobic chamber for 24 h (Figure 5A). Even after 3 h of incubation in an anaerobic chamber in this pre-reduced spent medium, cells were able to regrow rapidly (Figure 5C), similar to cells that had not been resuspended in spent medium (Figure 4B). These data suggest that respiration during transient growth and subsequent nutrient depletion in spent medium is the cause of growth defects and lysis.

## Discussion

Our data show that cells eventually adapt and expand at a slow rate in spent medium in a microfluidic flow cell (Figure 1C). This growth is consistent with their ability to remain metabolically active in stationary phase (Gefen et al., 2014) and remodel their proteome in the absence of carbon sources (Shi et al., 2021b). In this study, we present a model based on this growth potential that accounts for two dramatically different outcomes of starvation. On one hand, the steady maintenance of low levels of nutrients in a microfluidic flow cell that supports slow growth after growing cells are switched into spent medium means that the physiological state of the cell can be largely maintained. Cells are thus able to resume growth at approximately the same rate as prior to exposure to spent medium (Figure 2D), even after many hours (Figures 3B,C). Similar behavior results when growing cells are resuspended in water (Figure 5B), likely because the complete lack of nutrients in water preserves cellular physiology. On the other hand, if growing cells are switched into spent medium in a context that provides limited capacity for growth such as in a test tube, we propose that respiration produces damage during growth that cells cannot repair after nutrients are fully depleted, resulting in extensive cell lysis and growth slowdown (Figures 4C–F). It is also possible that other factors correlated with oxygen availability, such as the energy produced by electron transport chain activity or the ability to sustain rapid growth, contribute to the development of protein aggregates.

By contrast with switching between carbon sources (Lambert and Kussell, 2014), a switch to spent medium has much less potential for dilution, and hence cells need not shift away from the optimal state for previous growth conditions. Indeed, when restored to fresh medium in a microfluidic flow cell, growth restarted at quantitatively the same level as if they had never experienced spent medium (Figure 2D). Nonetheless, the consequences of switches between spent and fresh media can be extensive, as evidenced by the massive lysis due to transient exposure of stationary-phase cells to fresh medium followed by resuspension in spent-medium in a test tube (Figure 4E). In particular, some cells exhibited puzzling oscillations between growth and shrinking (Figure 4F). Altogether, these findings indicate the necessity for cells to have adequate time to prepare for starvation, consistent with the adaptations in growth rate (Atolia et al., 2020), cell shape (Shi et al., 2021a), and gene expression (Magnusson et al., 2005) that occur multiple doublings before cells reach saturation.

Our findings highlight fundamental but often overlooked differences between growth in flow cells and in test tubes. In a microfluidic chamber, flow keeps the environment relatively steady, whereas any resources are depleted over time in a test tube. Furthermore, waste can accumulate in a test tube, whereas it is washed away in a microfluidic device. This distinction may have relevance for survival in marine environments, which have low nutrient density and may behave more like a flow cell due to fluid flow. Given the large differences between the physiological states of cells in microfluidics versus test tubes, underscored by the differential effects of transient exposure to fresh medium in the two contexts (Figures 3–5), our results highlight the importance of considering nutrient and energy depletion as a continuum rather than a single state. Indeed, ATP depletion through dinitrophenol treatment caused immobilization of large particles in the cytoplasm of *E. coli* (Parry et al., 2014) whereas nutrient removal did not (Shi et al., 2021b). In the future, a more comprehensive exploration of the transcriptomic, metabolomic, and physical state of cells that have reached stationary phase in various manners may reveal the general and specific features of starvation.

Complementing previous studies of osmotic-shock oscillations (Rojas et al., 2014; Zhou et al., 2015; Rojas et al., 2017, 2018), our work highlights the informative nature of periodic fluctuations about cellular adaptation. Other relevant transitions of variables that can be rapidly adjusted such as temperature, pH, and oxygen levels should also be considered in the future. It is unknown whether most cells in microbial ecosystems such as the mammalian intestine are growing quickly or in stationary phase; likely they experience frequent oscillations in nutrient conditions, and hence comparing their response in relatively constant environments versus when cells can modify the environment should help to reveal their robustness to such fluctuations.

## Materials and methods

### Strain culturing

All cultures were grown in 3 ml of filter-sterilized LB at 37°C in glass test tubes with constant shaking at 225 rpm, in aerobic conditions except where indicated. Cultures were started at an OD of 0.1 with cells from a 5–7 h culture inoculated from a frozen stock (Figures 1, 2, 4, 5), or were inoculated from a frozen stock and grown overnight before being diluted 1:200 and grown for the specified interval (Figure 3). Spent medium was isolated by spinning down (at 4000 *g* for 5 min) and filtering a culture that had been grown for 17 h (Figures 1, 2, 4, 5) or 16 h (Figure 3) with a 0.22-μm polyethersulfone filter (Millex-GP SLGP033RS). The time point (16 h or 17 h) was determined by imaging cells on agarose pads made with fresh LB every hour starting at 12 h, and selecting the last time point without substantial heterogeneity in regrowth due to the onset of dormancy (Cesar et al., 2022). This time varied slightly between media batches.

### Measurements of population growth

Growth curves were obtained using an Epoch 2 Microplate Spectrophotometer (Biotek Instruments, Vermont). The plate reader went through 15-min cycles of incubation at 37°C, shaking linearly for 145 s, and then absorbance measurements (wavelength 600 nm, 25 flashes, 2-ms settle between flashes).

### Single-cell imaging on agarose pads

One microliter of cells was diluted 1:200 with fresh medium and spotted onto a pad of 1% agarose with LB, spent medium, or water as indicated, and imaged on a Nikon Eclipse Ti-E inverted fluorescence microscope with a 100X (NA 1.40) oil-immersion objective (Nikon Instruments). Phase-contrast and epifluorescence images were collected on a DU885 electron-multiplying CCD camera (Andor Technology) or a Neo sCMOS camera (Andor Technology) using μManager v. 1.4 (Edelstein et al., 2010). Cells were maintained at 37°C during imaging with an active-control environmental chamber (Haison Technology).

### Microfluidic flow cell experiments

For experiments initialized with stationary phase cells, cultures were grown for 17 h before being loaded into a microfluidic flow cell (CellASIC). For experiments initialized with exponentially growing cells, overnight cultures grown in LB were diluted 1,000-fold into LB and incubated at 37°C until the cells were in log phase. These cultures were diluted 100-fold into pre-warmed medium and loaded into a microfluidic flow cell (CellASIC). To ensure that cells were growing exponentially, the flow cell was incubated for an additional 1 h in the microscope environmental chamber, which was preheated to 37°C, before cells were imaged. Before loading cells into the imaging chamber of the flow cell, the chamber was primed with growth medium using the ONIX microfluidic perfusion platform (CellASIC). While imaging, fresh or spent medium was perfused through the flow cell as indicated.

### Image analysis

The MATLAB (MathWorks, Natick, MA, USA) image processing code *Morphometrics* (Ursell et al., 2017) was used to segment cells and to identify cell outlines from phase-contrast microscopy images. Cell area was used for growth rate measurements, as follows. Segmented cell outlines were used to calculate area *A_i_* at each time point *t_i_*, and the instantaneous growth rate 1/*A dA*/*dt* at time *t_i_* was estimated as $1/A_{i}\left(A_{i+1}-A_{i}\right)/\left(t_{i+1}-t_{i}\right).$ Occasionally growth rate increased or decreased in magnitude by several fold for only a single time point due to focus issues; those time points were removed from the dataset.

For Figure 2C, mCherry fluorescence intensity was quantified for all pixels within a given cell contour and the number of pixels above a threshold (350) was computed. The threshold was determined via manual inspection.

## Data availability statement

The original contributions presented in the study are included in the article/Supplementary material, further inquiries can be directed to the corresponding author.

## Author contributions

SC and KH designed the research. SC and JS performed the research. SC, JS, and KH analyzed the data and wrote the manuscript. All authors contributed to the article and approved the submitted version.

## Funding

The authors acknowledge funding from a National Science Foundation Graduate Research Fellowship (to SC), the Allen Discovery Center at Stanford on Systems Modeling of Infection (to KH), and NSF grant EF-2125383 (to KH). KH is a Chan Zuckerberg Biohub Investigator.

## Conflict of interest

The authors declare that the research was conducted in the absence of any commercial or financial relationships that could be construed as a potential conflict of interest.

## Publisher’s note

All claims expressed in this article are solely those of the authors and do not necessarily represent those of their affiliated organizations, or those of the publisher, the editors and the reviewers. Any product that may be evaluated in this article, or claim that may be made by its manufacturer, is not guaranteed or endorsed by the publisher.

## References

[ref1] Atolia E. Cesar S. Arjes H. A. Rajendram M. Shi H. Knapp B. D.. (2020). Environmental and physiological factors affecting high-throughput measurements of bacterial growth. mBio 11, e01378–e01320. doi: 10.1128/mBio.01378-20 33082255 PMC7587430

[ref2] Basan M. Honda T. Christodoulou D. Horl M. Chang Y. F. Leoncini E.. (2020). A universal trade-off between growth and lag in fluctuating environments. Nature 584, 470–474. doi: 10.1038/s41586-020-2505-4, PMID: 32669712 PMC7442741

[ref3] Cesar S. Willis L. Huang K. C. (2022). Bacterial respiration during stationary phase induces intracellular damage that leads to delayed regrowth. Iscience 25:103765. doi: 10.1016/j.isci.2022.103765, PMID: 35243217 PMC8858994

[ref4] Edelstein A. Amodaj N. Hoover K. Vale R. Stuurman N. (2010). Computer control of microscopes using microManager. Curr. Protoc. Mol. Biol. Chapter 14 92:Unit14.20. doi: 10.1002/0471142727.mb1420s92, PMID: 20890901 PMC3065365

[ref5] Erickson D. W. Schink S. J. Patsalo V. Williamson J. R. Gerland U. Hwa T. (2017). A global resource allocation strategy governs growth transition kinetics of *Escherichia coli*. Nature 551, 119–123. doi: 10.1038/nature24299, PMID: 29072300 PMC5901684

[ref6] Farrell M. J. Finkel S. E. (2003). The growth advantage in stationary-phase phenotype conferred by *rpoS* mutations is dependent on the pH and nutrient environment. J. Bacteriol. 185, 7044–7052. doi: 10.1128/JB.185.24.7044-7052.2003, PMID: 14645263 PMC296246

[ref7] Gefen O. Fridman O. Ronin I. Balaban N. Q. (2014). Direct observation of single stationary-phase bacteria reveals a surprisingly long period of constant protein production activity. Proc. Natl. Acad. Sci. U. S. A. 111, 556–561. doi: 10.1073/pnas.1314114111, PMID: 24344288 PMC3890821

[ref8] Gray D. A. Dugar G. Gamba P. Strahl H. Jonker M. J. Hamoen L. W. (2019). Extreme slow growth as alternative strategy to survive deep starvation in bacteria. Nat. Commun. 10:890. doi: 10.1038/s41467-019-08719-8, PMID: 30792386 PMC6385201

[ref9] Grimm J. Shi H. Wang W. Mitchell A. M. Wingreen N. S. Huang K. C.. (2020). The inner membrane protein YhdP modulates the rate of anterograde phospholipid flow in *Escherichia coli*. Proc. Natl. Acad. Sci. U. S. A. 117, 26907–26914. doi: 10.1073/pnas.2015556117, PMID: 33046656 PMC7604412

[ref10] Iyer S. Le D. Park B. R. Kim M. (2018). Distinct mechanisms coordinate transcription and translation under carbon and nitrogen starvation in *Escherichia coli*. Nat. Microbiol. 3, 741–748. doi: 10.1038/s41564-018-0161-3, PMID: 29760462

[ref11] Lambert G. Kussell E. (2014). Memory and fitness optimization of bacteria under fluctuating environments. PLoS Genet. 10:e1004556. doi: 10.1371/journal.pgen.1004556, PMID: 25255314 PMC4177670

[ref12] Magnusson L. U. Farewell A. Nystrom T. (2005). ppGpp: a global regulator in *Escherichia coli*. Trends Microbiol. 13, 236–242. doi: 10.1016/j.tim.2005.03.008 15866041

[ref13] Malinverni J. C. Silhavy T. J. (2009). An ABC transport system that maintains lipid asymmetry in the Gram-negative outer membrane. Proc. Natl. Acad. Sci. U. S. A. 106, 8009–8014. doi: 10.1073/pnas.0903229106, PMID: 19383799 PMC2683108

[ref14] Nguyen J. Fernandez V. Pontrelli S. Sauer U. Ackermann M. Stocker R. (2021). A distinct growth physiology enhances bacterial growth under rapid nutrient fluctuations. Nat. Commun. 12:3662. doi: 10.1038/s41467-021-23439-8, PMID: 34135315 PMC8209047

[ref15] Parry B. R. Surovtsev I. V. Cabeen M. T. O'hern C. S. Dufresne E. R. Jacobs-Wagner C. (2014). The bacterial cytoplasm has glass-like properties and is fluidized by metabolic activity. Cells 156, 183–194. doi: 10.1016/j.cell.2013.11.028, PMID: 24361104 PMC3956598

[ref16] Pu Y. Li Y. Jin X. Tian T. Ma Q. Zhao Z.. (2019). ATP-dependent dynamic protein aggregation regulates bacterial dormancy depth critical for antibiotic tolerance. Mol. Cell 73:e144, 143–156.e4. doi: 10.1016/j.molcel.2018.10.022 30472191

[ref17] Rojas E. R. Billings G. Odermatt P. D. Auer G. K. Zhu L. Miguel A.. (2018). The outer membrane is an essential load-bearing element in Gram-negative bacteria. Nature 559, 617–621. doi: 10.1038/s41586-018-0344-3, PMID: 30022160 PMC6089221

[ref18] Rojas E. R. Huang K. C. Theriot J. A. (2017). Homeostatic cell growth is accomplished mechanically through membrane tension inhibition of cell-wall synthesis. Cell Syst. 5:e576, 578–590.e6. doi: 10.1016/j.cels.2017.11.005 PMC5985661 29203279

[ref19] Rojas E. Theriot J. A. Huang K. C. (2014). Response of *Escherichia coli* growth rate to osmotic shock. Proc. Natl. Acad. Sci. U. S. A. 111, 7807–7812. doi: 10.1073/pnas.1402591111, PMID: 24821776 PMC4040581

[ref20] Sharma U. K. Chatterji D. (2010). Transcriptional switching in *Escherichia coli* during stress and starvation by modulation of sigma activity. FEMS Microbiol. Rev. 34, 646–657. doi: 10.1111/j.1574-6976.2010.00223.x, PMID: 20491934

[ref21] Shi H. Colavin A. Bigos M. Tropini C. Monds R. D. Huang K. C. (2017). Deep phenotypic mapping of bacterial cytoskeletal mutants reveals physiological robustness to cell size. Curr. Biol. 27:e3414, 3419–3429.e4. doi: 10.1016/j.cub.2017.09.065 29103935

[ref22] Shi H. Hu Y. Odermatt P. D. Gonzalez C. G. Zhang L. Elias J. E.. (2021a). Precise regulation of the relative rates of surface area and volume synthesis in bacterial cells growing in dynamic environments. Nat. Commun. 12:1975. doi: 10.1038/s41467-021-22092-5, PMID: 33785742 PMC8009875

[ref23] Shi H. Westfall C. S. Kao J. Odermatt P. D. Anderson S. E. Cesar S.. (2021b). Starvation induces shrinkage of the bacterial cytoplasm. Proc. Natl. Acad. Sci. U. S. A. 118:e2104686118. doi: 10.1073/pnas.2104686118 34117124 PMC8214708

[ref24] Simsek E. Kim M. (2019). Power-law tail in lag time distribution underlies bacterial persistence. Proc. Natl. Acad. Sci. U. S. A. 116, 17635–17640. doi: 10.1073/pnas.1903836116, PMID: 31427535 PMC6731627

[ref25] Sutterlin H. A. Shi H. May K. L. Miguel A. Khare S. Huang K. C.. (2016). Disruption of lipid homeostasis in the Gram-negative cell envelope activates a novel cell death pathway. Proc. Natl. Acad. Sci. U. S. A. 113, E1565–E1574. doi: 10.1073/pnas.1601375113, PMID: 26929379 PMC4801249

[ref26] Ursell T. Lee T. K. Shiomi D. Shi H. Tropini C. Monds R. D.. (2017). Rapid, precise quantification of bacterial cellular dimensions across a genomic-scale knockout library. BMC Biol. 15:17. doi: 10.1186/s12915-017-0348-8, PMID: 28222723 PMC5320674

[ref27] Zambrano M. M. Kolter R. (1996). GASPing for life in stationary phase. Cells 86, 181–184. doi: 10.1016/S0092-8674(00)80089-6, PMID: 8706122

[ref28] Zhou X. Halladin D. K. Rojas E. R. Koslover E. F. Lee T. K. Huang K. C.. (2015). Bacterial division. Mechanical crack propagation drives millisecond daughter cell separation in *Staphylococcus aureus*. Science 348, 574–578. doi: 10.1126/science.aaa1511, PMID: 25931560 PMC4864021

