# Supplementary material for: Cellular memory of rapid growth is sensitive to nutrient depletion during starvation
Source: Front Microbiol. 2022 Nov 21;13:1016371. doi: 10.3389/fmicb.2022.1016371 (PMC9719910; doi:10.3389/fmicb.2022.1016371)
Supplement: Supplementary file 3 [file Table_1.docx]

**Supplementary Table**

| Strain | Genotype | Source/reference |
| --- | --- | --- |
| *E. coli* MG1655 | *Escherichia coli* K-12 wild-type | CGSC #6300 |
| KC1193 | MG1655, attHK::P_lac_-dsbA^ss^-mCherry, pZS21-GFP | (Sutterlin et al., 2016) |

**Table S1: Strains used in this study.**
